# Clinical and Radiologic Outcomes after Anatomical Total Shoulder Replacement Using a Modular Metal-Backed Glenoid after a Mean Follow-Up of 5.7 Years

**DOI:** 10.3390/jcm11206107

**Published:** 2022-10-17

**Authors:** Emil Noschajew, Felix Rittenschober, Harald Kindermann, Reinhold Ortmaier

**Affiliations:** 1 Department of Orthopedic Surgery, Ordensklinikum Barmherzige Schwestern Linz, Vinzenzgruppe Center of Orthopedic Excellence, Teaching Hospital of the Paracelsus Medical University, 5020 Salzburg, Austria; 2 Department of Marketing and Electronic Business, University of Applied Sciences Upper Austria, Campus 4400 Steyr, 4600 Wels, Austria

**Keywords:** clinical outcome, metal back glenoid, midterm results, prosthesis, radiologic outcome, shoulder

## Abstract

Background: Glenoid wear is a common complication of anatomical total shoulder arthroplasty (aTSA) with a metal-backed glenoid (MBG), and the clinical and radiological results of historical implants are poor. The aim of this work was to evaluate the clinical and radiological results of 25 participants as well as the longevity after implantation of an anatomic shoulder prosthesis with a recent, modular cementless flat metal-backed glenoid component after a mean follow-up of 5.7 years. Methods: Clinically, the Simple Shoulder Test (SST), UCLA Activity Score (UCLA), and Constant Murley Score (CMS) were evaluated. Radiographically, the radiolucent lines (RLs), humeral head migration (HHM), and lateral glenohumeral offset (LGHO) were assessed. Survival was calculated with Kaplan–Meier curves and life-table analysis. Results: The mean CMS at follow-up was 46.2 points (range: 14–77; SD: 19.5). In terms of the SST score, the average value was 6.5 points (range: 1–10; SD: 3.5). The UCLA activity score showed a mean value of 5.9 points (range: 1–9; SD: 2.1). There were 17 revisions after a mean follow-up of 68.2 months (range: 1.8–119.6; SD: 27.9). HHM occurred in every patient, with a mean measurement of 6.4 mm (range: 0.5–13.4; SD: 3.9; *p* < 0.0001). The mean LGHO between the initial postoperative and follow-up images was 2.6 mm (range: 0–4.0; SD: 1.5; *p* < 0.0001). RLs were found in 22 patients (88%) around the glenoid and in 21 patients (84%) around the humeral head prosthesis. Conclusion: The clinical and radiographic outcomes after metal-backed glenoids were poor at 2.2 to 8.4 years of follow-up. We determined devastating survival in the majority of cases (68%), with mostly inlay wear (71%) as the main reason that led to revision surgery. The use of metalback genoids cannot be recommended based on the data of this study.

## 1. Introduction

Over the past decades, the total number of implanted TSAs has increased significantly, and this trend continues [1,2]. Despite a huge overhand of reverse total shoulder implants (RSA), the main indication for aTSA is indicated in patients with primary osteoarthritis (OA) with an intact rotator cuff (RTC) and no severe glenoid retroversion, biconcavity, or bone defect and younger age [3]. In this patient group, aTSA was still the implant of choice.

Longevity and low complication rates are crucial for patients, especially younger individuals. For aTSA survival, the glenoid component represents the weak link [4,5,6]. There are two types of glenoid components: cemented all-polyethylene glenoids (PEGs) and MBGs.

The usual pick for aTSA is the cemented all-PEG. However, high rates of glenoid component loosening and wear are reported in cemented all-PEGs [6]. The first attempts to improve the stability of glenoid components have led to the development of metal-backed implants. As a rule, MBG components consist of the “metal back” itself and a polyethylene (PE) component that articulates with the humeral head component. This creates a further contact surface between two different materials with possible complications, e.g., dissociation of the two parts or abrasion of the components. Additionally, these glenoids can increase the width of the two components or reduce the PE content and may stress shield the underlying bone due to primary stable fixation [7]. The results of historical metalback glenoids in the literature are rather poor, and based on a systematic review of Papadonikolakis and Matsen carried out in 2014, it was determined that MBGs are not advisable as they have higher failure rates [6]. Following the success of reverse prostheses, the development of modular MBG implants is currently attracting renewed interest. These implants have the potential to be used for both anatomical and reverse shoulder endoprostheses. Revision surgery should theoretically be less complicated as the glenoid baseplate does not require removal [8]. The purpose is thus to lower glenoid component loosening rates and raise the possibility of revising the implant via RSA due to the modularity of most implants. Despite concerning reports of high complication rates of MBGs in aTSA, newer designs promise to lower the complication rate and yield better results with the possibility of converting the prosthesis very easily to a reverse implant if necessary [8]. In this study, we evaluated the clinical and radiological results, as well as the survival rate of the aTSA with a modular cementless flat MBG.

## 2. Materials and Methods

### 2.1. Study Population

All subjects gave their informed consent for inclusion before participating in the study. The study was conducted in accordance with the Declaration of Helsinki, and the protocol was approved by the local Ethics Committee of the state of Upper Austria (Study number 1167/2020). The case number consisted of 25 patients (15 women) who underwent shoulder arthroplasty in the period from 01/2009 to 07/2020. Included in the study were all patients who received an aTSA with a flat MBG in the specified period. The indication for implantation was an intact RTC without severe fatty infiltration (Fuchs grade ≤ 2) as well as radiographically determined omarthrosis, which was accompanied by severe pain in the shoulder joint and restricted movement of the affected arm and glenoid morphology according to Walch A1, 2 and B1 [9,10]. The exclusion criteria for performing aTSA included a full-thickness RTC tear and/or fatty infiltration of the RTC (Fuchs grade > 2), glenoid morphology according to Walch B2, B3, C, and D.

The minimum follow-up time from prosthesis implantation to the last reevaluation was 24 months, with a mean follow-up time of 68.6 months (range: 25.9–100.7). All patients were required to have pre- and postoperative radiographic images of the operated shoulder. Exclusion criteria for participating in the study were neurologic abnormalities or inability to fulfill the study requirements.

### 2.2. Data Collection and Assessment

Clinically, the postoperative Constant Murley Score (CMS), UCLA-Score, and the Simple Shoulder Test (SST) were assessed at the final follow-up. Radiologically, every patient had preoperative X-rays in two planes (anterior-posterior (AP) and axillary or y-view) and MRI or CT. CT and MRI were used for the classification of the preoperative glenoid morphology, according to Walch et al. [10], and RTC degeneration, according to Fuchs et al. [9]. Immediately postoperatively and at final follow-up, all patients received at least an X-ray in two planes (AP and axillary or y-view). 

**Figure 1:**
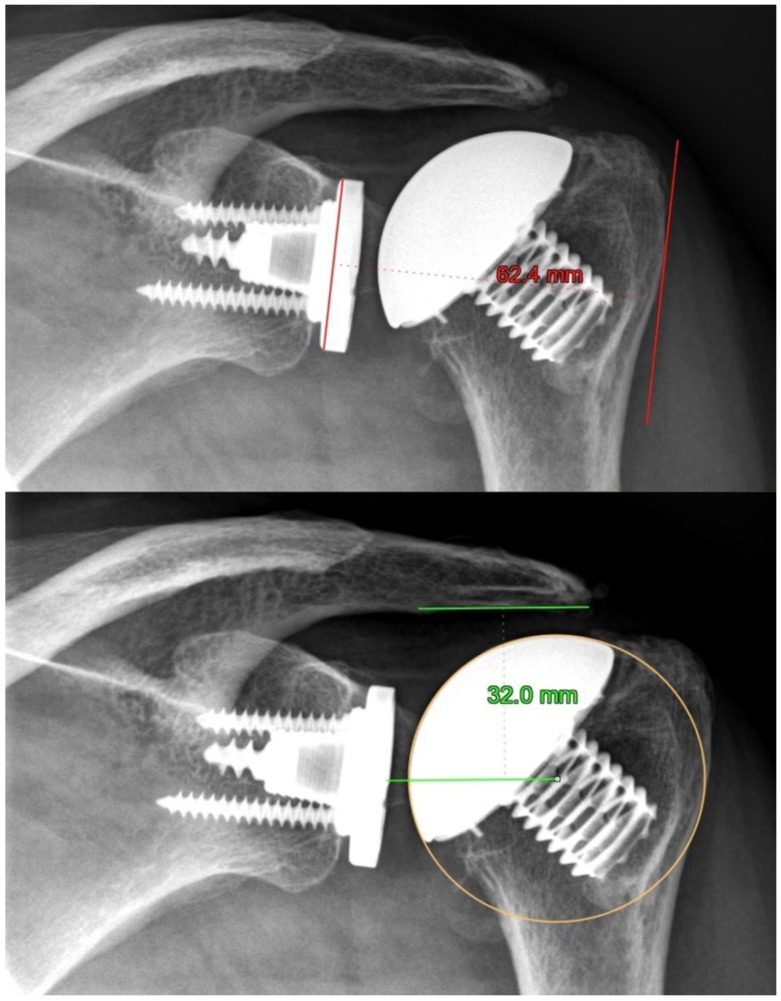
Postoperative radiographs of a left shoulder demonstrate the measurement method of the lateral glenohumeral offset (above X-ray image) and the humeral head migration (below X-ray image).

The postoperative X-ray images were calibrated over the known head size of the implanted humeral head. Radiolucent lines (RLs) around the humeral and glenoid components were assessed from the postoperative X-rays. Postoperative X-rays were also used to evaluate the center of rotation (COR). Postoperative radiographs were needed to measure the humeral head migration (HHM) and lateral glenohumeral offset (LGHO). HHM was measured via the smallest distance between the COR and the dense cortical bone marking the underside of the acromion. The difference between immediately postoperative AP X-rays and AP X-rays at final follow-up was calculated. The COR was determined as described by Alolabi et al. [11]. The debridement of the PE was measured using the LGHO as a difference (millimeter) of LGHO from immediately postoperative AP X-rays and LGHO from AP X-rays at final follow-up, which was determined by the distance from the medial edge of the baseplate to the most lateral point of the greater tuberosity (Figure 1).

To evaluate RLs around the glenoid, the Lazarus scoring system, originally described for pegged glenoid components, was applied [12]. To evaluate the RLs around the humeral component in our study, the AP radiographs were assessed, and the axillary view was taken by dividing the implant-bone interface into three different sections. 

**Figure 2:**
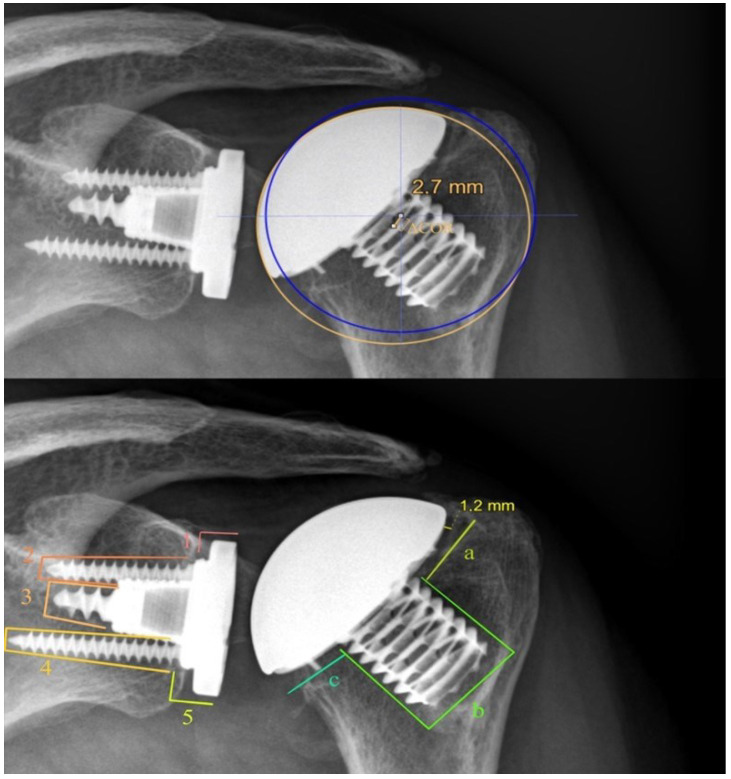
Postoperative radiographs of the left shoulder. The above X-ray image shows the anatomical circle (blue) and the implant-matched circle (orange), according to Alolabi et al. [11]. The distance between the two centers was measured (ΔCOR). In the X-ray image below, an assessment of radiolucent lines (RLs) for the glenoid and humeral components is shown. Glenoid RLs were quantified in 5 zones (1–5) considering their thickness, while the humeral RLs were quantified in 3 different zones (a, b, and c). The Radiograph shows radiolucency in zone a.

Similarly, the radiolucent lines around the humeral component were assessed using eight distinct zones. For the humeral components, the analysis was based on the classification by Molé et al. [13] (Figure 2).

### 2.3. Statistical Analysis

For statistical analysis, a comparison was made between the values originally collected postoperatively and those collected at the follow-up examination. If the Shapiro–Wilk test did not obtain a normal distribution, the Wilcoxon test for paired samples was used instead. We also used descriptive statistics for data evaluation. The data were evaluated and compared using Origin Pro^®^ 9.0 (OriginLab Corp, Northampton, MA, USA) and SPSS^®^ 26.0 software (IBM Corp, Armonk, NY, USA). OriginLab^®^ was used to create a Kaplan–Meier plot for the endpoints defined as revision for conversion to RSA and revision for any reason. Furthermore, common statistical methods, such as the mean values, medians, effect sizes, ranges, and standard deviations (SDs), were used in this study.

## 3. Results

None of the patients were lost to the follow-up. The average age of the patients at the time of surgery was 64.8 years (SD: 11.0) and, on the day of the examination, 70.9 (SD: 8.7) years. Most of the subjects received the prosthesis on their left shoulder, comprising 13 patients (52%). Among them, 12 patients were right-handed. A total of 12 patients had prostheses implanted on the non-dominant side. In all patients, an Eclipse™ humeral prosthesis combined with a Universal Glenoid™ baseplate from Arthrex^®^ was implanted. The Univers 3D Metal Back is made of the Material Ti6Al4V. The Eclipse™ Humeral Head is made of the material CoCr, and the inlay is made of PE. The company of the tools, Arthrex Inc., is based in Naples, FL (34108-1945), USA. Details of the implants used in every patient were documented, and acceptable combinations between the size of the baseplate and humeral head in which the radii fit together properly were chosen for every patient. Table 1 shows patients’ demographics, implant details, preoperative glenoid morphology, RTC degeneration, and postoperative deviation from the native center of rotation.

### 3.1. Clinical Outcome

The total score of CMS on the implanted arm at follow-up was, on average, 46.2 points (range: 14–77; SD: 19.5) in 25 patients. Of those patients, the average total score on the unaffected arm was 75.4 points (range: 22–100; SD: 18.4). On the assessment day, the average score on the affected arm in the strength area was 3.8 points (SD: 3.7). The patients rated the subjective pain classification using the VAS ranging from 0 to 15, with an average of 9.7 (SD: 3.7) out of 15 points. Only one participant (4%) achieved complete freedom from pain. The mean score for everyday activities was 12.8 (range: 3–20; SD: 5.3). At the follow-up examination, the mean score in the mobility range was 19.9 (range: 4–38; SD: 9.9). The results of the SST in the surveys had a mean score of 6.5 (range: 1–10; SD: 3.5), with a median of 8. The UCLA score was, on average, 5.9 (range: 1–9; SD: 2.1), with the median lying at 5 points.

### 3.2. Radiologic Outcome

In 22 patients (88%), RLs around the glenoid component were found. In 21 (84%) patients, the humeral component showed RLs. The overall mean Lazarus grade for the glenoid component was 2.1 points (range: 0–4; SD: 1.1; *p* < 0.001; effect size: 2.0). There were only two patients with an RL thicker than 2 mm. In detail, there were three patients with a Lazarus grade of 0 (12%), three patients with a grade of 1 (12%), eight patients with a grade of 2 (32%), ten patients with a grade of 3 (40%), one patient with a grade of 4 (4%) and none with a grade of 5. The overall mean points given by the classification according to Molé et al. [13] were 3.4 (range: 0–7; SD: 2.17; *p* < 0.001; effect size: 1.6). Most patients had a score of 3, followed by a score of 0 and 6, each with the same number of frequencies. No patient had a score of 1, and only one had a score of 7.

Upward migration of the humeral head was observed in all of the patients. The mean difference between the HHM value of the initial postoperative radiograph and the latest follow-up was 6.4 mm (range: 0.5–13.4; SD: 3.9; *p* < 0.001; effect size: 1.6). In 7 study participants, the humeral head migrated more than 10 mm. On the other hand, only two patients had an HHM of less than 1 mm.

In 23 out of 25 cases, polyethylene wear was detected after a mean follow-up of 62.3 months. For LGHO, the mean difference between the first postoperative radiograph and the last follow-up was 2.6 mm (range: 0–4.0; SD: 1.5; *p* < 0.001; effect size: 1.7). In most patients (9), the inlay wore between 2 and 4 mm. On the other hand, only four patients had an LGHO difference of 1 to 2 mm.

### 3.3. Complications and Revisions

**Figure 3:**
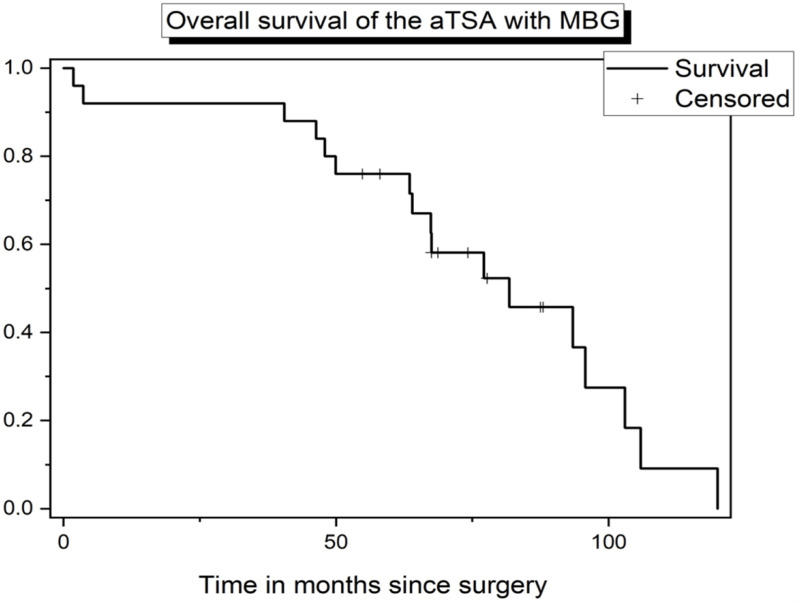
Kaplan–Meier plot depicting survival of the aTSA with MBG from implant revision for any reason among the study population.

Seventeen patients (8 women) were revised, mostly because of polyethylene wear. In patients undergoing revision surgery for any reason, the mean age at implantation of the anatomic prosthesis was 63.3 years (range: 45–80; SD: 10.5), and at revision, the average age was 68.8 years (range: 51–84; SD: 9.6). Among our study group, the probability of prosthesis survival was 32% (17 revisions) after a mean follow-up of 68.2 months (range: 1.8–119.6; SD: 27.9). In 12 cases (71%), PE wear was the most prevalent reason for revision surgery. Three patients had RTC injuries, and one patient had glenoid loosening as the cause of the revision. Only one patient developed a wound infection after surgery, resulting in the need for revision. Figure 3 shows the overall implant survival curve of our study. There, after around 75 months, the median has been reached. Afterward, the revision cases occurred more frequently in less amount of time. Censored were all patients on their last follow-up time who did not undergo revision surgery. In the graph, it can be seen that the first revision occurred quite early, after 1.8 months. The last revision occurred after 119.6 months. The graph shows that the first half of the revisions took 3/4 of the total time span. In contrast, most of the revisions were done after the midpoint of the timeline.

**Figure 4:**
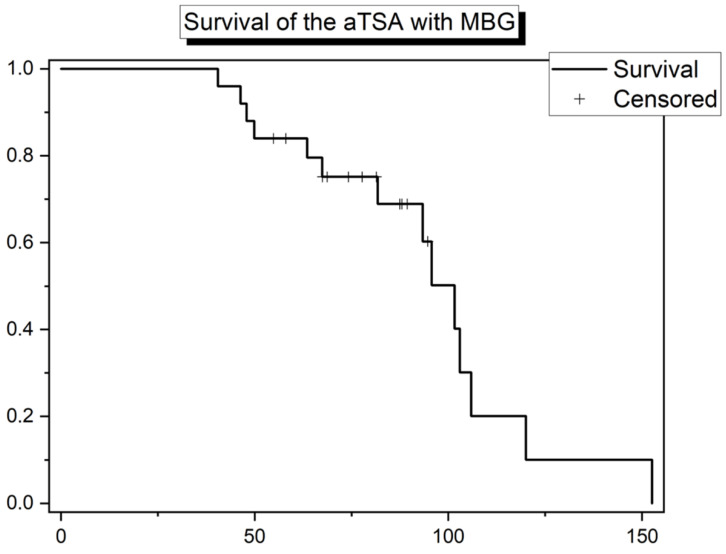
Kaplan–Meier plot depicting survival of the aTSA with MBG from implant revision for conversion to RSA among the study population.

Altogether, thirteen patients, seven of whom were female, were converted to RSA. Out of the 13 revision cases, there were different reasons for conversion to an RSA. With 85% (11 cases), the most common indication for revision was polyethylene wear. The remaining 2 cases had secondary RTC tears as reasons. In all of them, an explantation of the Eclipse™ implant and switch to Arthrex Reverse TSA was performed. The mean time to revision for conversion to RSA was 80.7 months (range: 40.5–152.6; SD: 30.9), whereas the mean age of the patients at revision was 71.2 years (range: 61–84; SD: 7.9). Figure 4 shows the Kaplan–Meier survival curve from conversion to RSA. The survival rate free of revision for conversion to RSA was 48% at 80.7 months. After approximately 85 months, half of the patients got conversion surgery to RSA. The median of revisions came in later than in the first Kaplan–Meier curve. The first conversion to RSA happened after 40.5 months and the last much later at 152.6 months, as seen in the Kaplan–Meier curve (Figure 4). 

**Figure 5:**
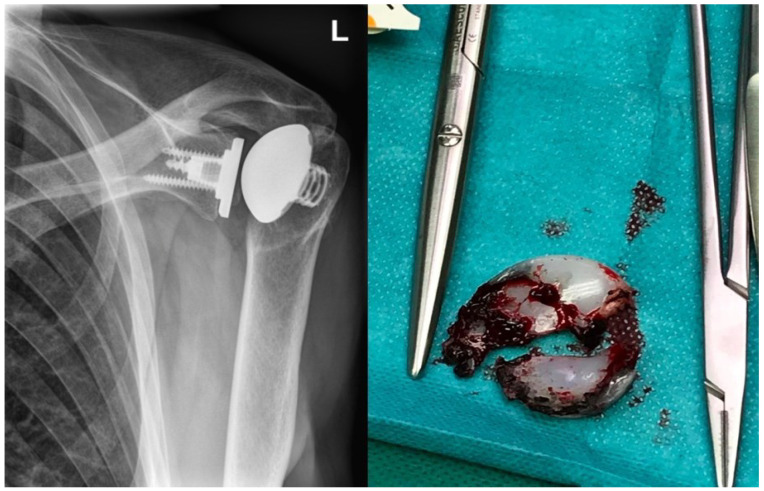
The image on the left side shows an X-ray taken shortly before a revision operation for conversion to RSA, demonstrating inlay abrasion with a tilt of the humeral head towards the metal-back as a direct sign of inlay abrasion and loosening of the humeral component with varus-tilt due to osteolysis and PE wear. The image on the right side presents an intraoperative situs of the inlay after removal.

The time interval between the first and last revision is described in Figure 4 as considerably higher. Figure 5 shows a radiograph taken right before a revision for conversion to RSA, highlighting both PE wear and humeral implant loosening with varus-tilting.

## 4. Discussion

Anatomical shoulder arthroplasty is an effective method for treating degenerative joint diseases if the bone substance is enough and the RTC is intact [14]. Nevertheless, compared to knee or hip replacements, TSAs have a relatively short lifespan, averaging ten years [15]. Therefore, each component of the shoulder prosthesis should be well chosen, aiming for the longest possible survival. Any part of the prosthesis can lead to revision surgery if it is not harmonized with the other components or is flawed.

At a mean follow-up of 68.2 months (range: 1.8–119.6; SD: 27.9), complete revision in our study cohort was required in 17 patients (68%), and 71% of shoulders undergoing revision (12 of 17) had PE wear as the main reason. These results were similar to findings from a study by Gauci et al. [16], in which a total of 26 out of 69 shoulders were revised, including 16 out of 26 shoulders in the MBG group. After a follow-up period of 12 years, the survival of the implants was 24% (SD: 0.10) for the metal-backed components. PE wear with metal-on-metal contact, RTC deficiency, and instability accounted for revision in the MBG group [16]. Gauci et al. [16] had a similar patient number, indications for revision, and survival rates. However, their follow-up period was longer than ours, so their findings may indicate what we have to deal with in the future, namely, a decrease in the survival of the implants. Over time, the chances of degenerative changes in the bone and deterioration of the inlay increase due to extended use of the shoulder joint after implantation of the prosthesis.

Another study conducted by Boileau et al. [17], also showed with 46% a very poor survival rate in 165 TSAs with 2 to 16 years of follow-up and a mean age of 68 years. These patients were diagnosed with primary OA and then treated with aTSA using an uncemented MBG component. The outcome for the survival rate free of revision was 46% at 12 years, with the endpoint for the survival curve defined as either complete or partial revision. Of the study population, 61 patients, or 37%, had undergone revision surgery after a mean follow-up of 8.5 years, 49 of whom had evidence of PE inlay wear [17]. It is worth mentioning that in our study, a similar rate of revisions was caused by PE wear. For the survival rates in our study, the longevity of the prostheses until revision surgery was reported. The mean follow-up was greater than ours, and our study’s indication for aTSA with MBG was also mainly OA. The revisions of the patients in our study came in earlier. However, this study has a higher patient coverage.

To date, with 570 metal-backed TSAs, the most extensive series show low survival rates with MBG implants in aTSA with 95 revisions from a total of 121 accounting for metal-backed prostheses after 15 years [18]. This shows that prostheses with MBGs lead to increasing revision rates over time, rendering MBGs inadvisable for long-term use. A systematic literature search was conducted by Papadonikolakis and Matsen [19] regarding papers stating radiographic leakage, loosening, or revision of the glenoid component in aTSAs that had been carried out in patients of all ages and with any diagnosis. They found that when comparing 1571 MBG and 3035 full-PEG implants, the revision rate was more than three times higher for MBG components (14%) than for full-PE components (3.8%), according to the authors’ findings. As many as 77% of revisions of full-PE components were due to loosening, whereas 62% of revisions of MBG components occurred due to other causes, such as PE wear, metal wear, component dissociation or fracture, screw fracture, and RTC tear [20]. Our age distribution correlates with similar results of specific studies published by Gauci et al. [16] and Taunton et al. [21]. Age could also play a considerable part in the results since older patients may have a lower functional demand than younger patients or tend to have higher rates of RTC deficiency, which correlates with patient age [22]. It has been argued that, in comparison to older patients, young patients have higher functional demands and higher expectations of enhanced capacity for social interaction, participation in sports, and exercise [23].

The mean age of our patients was 70.9 years. Similar mean patient age of 68 years was found in the study by Taunton et al. [21]. It was also found that at an average follow-up of 9.5 years, the five-year survival rate in this study was 79.9%, and the 10-year survival rate was 51.9%, leading the authors to express significant concern about the utilization of metal-backed, uncemented glenoid components [21,24]. 

In our study, the objective and subjective clinical results were poor. The clinical outcomes of our study were, in general, worse than those in similar studies on the topic of aTSA with MBG, especially for CMS. The flat profile design of our MBG and the screws with a small diameter that we used could contribute to the worse outcome, as not all studies compared had a flat MBG or utilized screws with a small diameter. Altogether, with the ADL score, which was on average 12.8 (64%), the most important domains for patients’ quality of life were not as poor as might be expected. This could be mainly due to the advanced age of the patients and the relatively inactive life they lead. Almost all study participants were retirees who did not require high mobility. A similar negative result was found in a study by Clement et al. [25]. Therein, 49 shoulders with metal-backed glenoids of 39 patients had an average CMS of 33.5 after a minimum follow-up of 132 months. However, no patient was able to abduct their arm to 90 degrees pre- or postoperatively [25]. A study by Fucentese et al. [26] that examined the clinical and radiographic outcomes associated with the use of an uncemented soft-metal-backed glenoid component found a CMS of 65.9 in 22 patients after a mean follow-up of 50 months [26]. The study from Gauci et al. [16] had a CMS of 64, similar to the one from Fucentese et al. [26], which was found in 7 shoulders with MBG from 23 MBG prostheses, of which 16 were lost to follow-up, at a mean follow-up of 10.3 years [16]. In 2017, Kany et al. [27] noted a mean CMS of 56.6 and a mean SST of 6.7 points in their study of 14 TSAs with MBG [27]. This implies a poor outcome in MBG prostheses, which was also apparent in our case. Another study by Kany et al. [28] found a mean CMS score of 60 in a total of 26 cases, 16 of whom had TSA with MBG and a mean SST score of 8 [28]. The study by Kany et al. [28] had a similar patient outcome but a moderately better CMS and SST score than ours. 

The radiological results are in concordance with the clinical results of our study. These radiographic results reaffirm the poor clinical scores. The radiological results correlate with the clinical results.

In our study, there was a huge difference in RLs from the first postoperative radiographic control to the follow-up. At follow-up, 22 patients (88%) had one or more RLs on the glenoid, and 21 patients (84%) had RLs on the humeral components, whereas none were found at the first postoperative admission. In contrast, the study by Boileau et al. [29] showed RLs in only 5 of a total of 20 shoulders with an MBG after a mean follow-up of 38.4 months [29]. For the humeral component, Gallacher et al. [14] analyzed a total of 100 shoulders from 86 patients with a mean follow-up of 35.4 months (range 24–76 months). The study found that 12% had incomplete RLs, and 4% had complete RLs [14]. Magosch et al. [30] conducted a study of 48 TSA-implanted patients with MBG who were clinically and radiologically followed up with a mean of 49 months. They found in total 4 cases of incomplete RLs, two cases with under 1 mm of thickness, and 2 cases with RLs<2 mm. As in our study, they did not find glenoid component loosening in their cases. However, we had more cases with RLs using the same prosthesis type as Magosch et al. [30]. In summary, 8 cases from their study required revision [30,31]. Unlike other studies, ours had a large number of RLs. The high amount of PE wear could be responsible for this. However, none of the MBGs at the follow-up were loose despite RLs and rarefication due to PE wear. Furthermore, in the literature, it has been reported that the prevalence of RLs in TSA ranges from 22% to 95%, and they occur in both types of glenoids, whether they are cemented or uncemented. However, evaluation of radiolucency from radiographs is prone to error, with standardization of patient position being difficult due to scapulothoracic mobility and individual anatomic differences. The lack of a standardized scoring system makes the comparison of findings challenging [32]. RLs rates were shown to be highly variable throughout studies, complicating comparisons of related factors.

HHM was detected on the AP radiographs in all patients within our study. Clinically, proximal humerus migration is important because it implicates a disturbance of normal glenohumeral kinematics from which advanced RTC disease is often a sign [33]. There was a mean difference between the HHM of the first postoperative radiograph and the last follow-up of 6.4 mm (range: 0.5–13.4; SD: 3.9; effect size: 1.6). This indicates a serious disturbance of normal glenohumeral kinematics. Montoya et al. [34] observed HHM in 8 of 53 patients after a mean follow-up of 64 months [34]. A comparison of HHM between our study and other findings in the literature reveals that HHM is more common in our study, but for the most part, the level of upward migration is rather moderate.

Regarding the LGHO, we see a similar pattern. Among 25 patients, 23 developed PE wear. In some patients, the MBG also showed signs of wear to some extent. The LGHO in our study had a mean difference between the first postoperative imaging and the follow-up of 2.6 mm (range: 0–4.0; SD: 1.5; effect size: 1.7). All patients who underwent revision had radiographic signs of wear of the glenoid component as well as superior HHM at follow-up. It has been shown by biomechanical and clinical studies that MBG implants used in aTSA have adverse effects on both the PE and the underlying glenoid bone [20]. In the future, finite element computational analysis (in silico) will allow implant designs to be tested and improved in advance. This could help reduce PE wear and therefore support the longevity of shoulder prostheses with an MBG [35,36].

Long-term studies of MBG implants and their LGHO measurements on radiographs are lacking, making comparative studies scarce. Nevertheless, this is an important issue that can be used to dispute the beneficial effect of MBG components in aTSA, as almost all individuals in the study population received HHM, RLs, and LGHO. Analysis of the results for clinical scores showed a similar trend as described above for radiological outcomes.

In summary, the propagated advantage of the new modular MBG components concerning prosthesis survivability cannot be confirmed in our study. The clinical results are consistent with the radiological results, which are also unacceptably poor.

The study conducted had some limitations. The number of patients was relatively low at 25. We did not include a control group for comparison purposes. A comparison group would be desirable, especially in view of the unusually high complication and revision rate. The results of the present study are also compared against old MBGs with known technical problems and a significantly longer follow-up. 

Due to the fact that angles and lengths are impacted by the location of the scapula and the humerus rotation, all radiographic measures are strongly reliant on the patient’s orientation during radiographic imaging. Radiographic imaging, even when standardized, can reveal massive differences. However, no patients were lost to follow-up. In addition, the same implant was used throughout the entire study population, with the same surgical technique being performed by two experienced shoulder surgeons.

## 5. Conclusions

In conclusion, considering all previously mentioned aspects, aTSAs with MBG provide poor clinical and radiological outcomes. Concerns from previous studies were fulfilled in that anatomic shoulder prostheses with an MBG present rapid inlay wear and have a low survival rate, reaching 32% in our study at 68.2 months. In the future, further long-term follow-up studies on modern MBGs need to be carried out, with more participants and the inclusion of a control group.

## Figures and Tables

**Table 1:** Patient demographics and implants.

Case	Age at Implantation	Gender	Indication	Prosthesis Side r: Right l: Left	Baseplate Size	Inlay Size	Humeral Head Size	Preoperative CT/MRI	Walch Classification of the Glenoid	Fuchs Classification of Rotator Cuff	ΔCOR in mm
**1**	74	f	OA	r	m	m	45	MRI	A1	SC: 1, SS: 1, IS: 1, TM: 2	2.7
**2**	58	f	OA	l	s	s	39	MRI	A2	SC: 1, SS: 2, IS: 2, TM: 1	1.5
**3**	73	m	OA	r	l	l	47	MRI	A2	SC: 1, SS: 2, IS: 1, TM: 1	4.0
**4**	78	f	OA	l	m	m	43	MRI	A2	SC: 1, SS: 2, IS: 1, TM: 1	1.8
**5**	67	m	OA	r	l	l	47	MRI	A2	SC: 1, SS: 2, IS: 1, TM: 1	1.5
**6**	59	f	OA	r	s	s	41	MRI	A2	SC: 1, SS: 1, IS: 1, TM: 1	2.8
**7**	70	f	pOA	r	m	m	47	MRI	A1	SC: 1, SS: 1, IS: 1, TM: 1	2.6
**8**	74	f	OA	r	l	l	49	MRI	A2	SC: 2, SS: 2, IS: 2, TM: 1	1.2
**9**	76	f	OA	l	m	m	43	MRI	A1	SC: 1, SS: 2, IS: 2, TM: 1	4.1
**10**	49	m	OA	r	m	m	43	MRI	A2	SC: 1, SS: 1, IS: 1, TM: 1	0.8
**11**	45	f	OA	r	m	m	43	CT	A1	SC: 1, SS: 1, IS: 1, TM: 1	1.6
**12**	68	f	OA	l	m	m	43	MRI	A1	SC: 1, SS: 1, IS: 1, TM: 1	1.7
**13**	56	m	OA	r	m	m	45	CT	A1	SC: 1, SS: 1, IS: 1, TM: 1	1.1
**14**	68	m	OA	l	m	m	43	MRI	A2	SC: 1, SS: 1, IS: 1, TM: 2	0.9
**15**	65	f	OA	l	s	s	41	MRI	A1	SC: 1, SS: 1, IS: 1, TM: 1	0.8
**16**	60	f	OA	r	m	m	41	MRI	A2	SC: 1, SS: 1, IS: 1, TM: 1	1.2
**17**	62	m	OA	l	s	s	41	MRI	A1	SC: 1, SS: 1, IS: 1, TM: 1	2.5
**18**	59	f	OA	l	s	s	43	MRI	A1	SC: 1, SS: 1, IS: 1, TM: 1	1.5
**19**	56	m	OA	l	m	m	47	CT	A1	SC: 1, SS: 1, IS: 1, TM: 1	1.6
**20**	63	m	OA	l	s	s	41	MRI	A1	SC: 1, SS: 1, IS: 1, TM: 1	2.2
**21**	61	f	OA	l	s	s	39	MRI	A1	SC: 1, SS: 1, IS: 1, TM: 1	1.0
**22**	80	f	pOA	r	m	m	47	MRI	A2	SC: 1, SS: 1, IS: 1, TM: 1	1.5
**23**	72	m	OA	l	l	l	43	MRI	A2	SC: 1, SS: 1, IS: 1, TM: 1	2.3
**24**	49	f	OA	l	m	m	43	MRI	A2	SC: 1, SS: 2, IS: 1, TM: 1	1.5
**25**	76	m	OA	r	l	l	43	MRI	A1	SC: 1, SS: 1, IS: 1, TM: 1	1.8

**Abbreviations:** SC: M. subscapularis, SS: M. supraspinatus, IS: M. infraspinatus, TM: M. teres minor, OA: osteoarthritis, pOA: posttraumatic OA.

## Data Availability

All data relevant to the study are included in the article. Details regarding where data supporting reported results can be asked at the following e-mail address: emil.noschaj@yahoo.de.

## References

[1] Kim S.H., Wise B.L., Zhang Y., Szabo R.M. (2011). Increasing incidence of shoulder arthroplasty in the United States. J. Bone Jt. Surg..

[2] Best M.J., Aziz K.T., Wilckens J.H., McFarland E.G., Srikumaran U. (2021). Increasing incidence of primary reverse and anatomic total shoulder arthroplasty in the United States. J. Shoulder Elb. Surg..

[3] Wiater J.M., Fabing M.H. (2009). Shoulder arthroplasty: Prosthetic options and indications. J. Am. Acad. Orthop. Surg..

[4] Bonnevialle N., Melis B., Neyton L., Favard L., Mole D., Walch G., Boileau P. (2013). Aseptic glenoid loosening or failure in total shoulder arthroplasty: Revision with glenoid reimplantation. J. Shoulder Elb. Surg..

[5] Somerson J.S., Hsu J.E., Neradilek M.B., Matsen F.A. (2018). Analysis of 4063 complications of shoulder arthroplasty reported to the US Food and Drug Administration from 2012 to 2016. J. Shoulder Elb. Surg..

[6] Kim D.M., Aldeghaither M., Alabdullatif F., Shin M.J., Kholinne E., Kim H., Jeon I.H., Koh K.H. (2020). Loosening and revision rates after total shoulder arthroplasty: A systematic review of cemented all-polyethylene glenoid and three modern designs of metal-backed glenoid. BMC Musculoskelet. Disord..

[7] Gustas-French C., Petscavage-Thomas J., Bernard S.A. (2018). Imaging of Shoulder Arthroplasties. AJR Am. J. Roentgenol..

[8] Castagna A., Garofalo R. (2019). Journey of the glenoid in anatomic total shoulder replacement. Shoulder Elb..

[9] Fuchs B., Weishaupt D., Zanetti M., Hodler J., Gerber C. (1999). Fatty degeneration of the muscles of the rotator cuff: Assessment by computed tomography versus magnetic resonance imaging. J. Shoulder Elb. Surg..

[10] Walch G., Badet R., Boulahia A., Khoury A. (1999). Morphologic study of the glenoid in primary glenohumeral osteoarthritis. J. Arthroplast..

[11] Alolabi B., Youderian A.R., Napolitano L., Szerlip B.W., Evans P.J., Nowinski R.J., Ricchetti E.T., Iannotti J.P. (2014). Radiographic assessment of prosthetic humeral head size after anatomic shoulder arthroplasty. J. Shoulder Elb. Surg..

[12] Lazarus M.D., Jensen K.L., Southworth C., Matsen F.A. (2002). The radiographic evaluation of keeled and pegged glenoid component insertion. J. Bone Jt. Surg..

[13] Molé D., Roche O., Riand N., Lévigne C., Walch G. (1999). Cemented glenoid component: Results in osteoarthritis and rheumatoid arthritis. Shoulder Arthroplasty.

[14] Gallacher S., Williams H.L.M., King A., Kitson J., Smith C.D., Thomas W.J. (2018). Clinical and radiologic outcomes following total shoulder arthroplasty using Arthrex Eclipse stemless humeral component with minimum 2 years’ follow-up. J. Shoulder Elb. Surg..

[15] Gregory T.M., Boukebous B., Gregory J., Pierrart J., Masemjean E. (2017). Short, Medium and Long Term Complications After Total Anatomical Shoulder Arthroplasty. Open Orthop. J..

[16] Gauci M.O., Bonnevialle N., Moineau G., Baba M., Walch G., Boileau P. (2018). Anatomical total shoulder arthroplasty in young patients with osteoarthritis: All-polyethylene versus metal-backed glenoid. Bone Jt. J..

[17] Boileau P., Moineau G., Morin-Salvo N., Avidor C., Godenèche A., Lévigne C., Baba M., Walch G. (2015). Metal-backed glenoid implant with polyethylene insert is not a viable long-term therapeutic option. J. Shoulder Elb. Surg..

[18] Fox T.J., Cil A., Sperling J.W., Sanchez-Sotelo J., Schleck C.D., Cofield R.H. (2009). Survival of the glenoid component in shoulder arthroplasty. J. Shoulder Elb. Surg..

[19] Papadonikolakis A., Matsen F.A.I. (2014). Metal-Backed Glenoid Components Have a Higher Rate of Failure and Fail by Different Modes in Comparison with All-Polyethylene Components: A Systematic Review. J. Bone Jt. Surg..

[20] Boileau P., Baba M., Moineau G., Morin-Salvo N., Avidor C., Godenèche A., Lévigne C., Walch G. (2016). Response to Katz et al: The weak link in metal-backed glenoid implants is the polyethylene. J. Shoulder Elb. Surg..

[21] Taunton M.J., McIntosh A.L., Sperling J.W., Cofield R.H. (2008). Total shoulder arthroplasty with a metal-backed, bone-ingrowth glenoid component. Medium to long-term results. J. Bone Jt. Surg..

[22] Khazzam M., Sager B., Box H.N., Wallace S.B. (2020). The effect of age on risk of retear after rotator cuff repair: A systematic review and meta-analysis. JSES Int..

[23] Rasmussen J.V., Olsen B.S. (2022). Previous surgery for instability is a risk factor for a worse patient-reported outcome after anatomical shoulder arthroplasty for osteoarthritis: A Danish nationwide cohort study of 3,743 arthroplasties. Acta Orthop..

[24] Schrumpf M., Maak T., Hammoud S., Craig E.V. (2011). The glenoid in total shoulder arthroplasty. Curr. Rev. Musculoskelet. Med..

[25] Clement N.D., Mathur K., Colling R., Stirrat A.N. (2010). The metal-backed glenoid component in rheumatoid disease: Eight- to fourteen-year follow-up. J. Shoulder Elb. Surg..

[26] Fucentese S.F., Costouros J.G., Kühnel S.P., Gerber C. (2010). Total shoulder arthroplasty with an uncemented soft-metal-backed glenoid component. J. Shoulder Elb. Surg..

[27] Kany J., Jose J., Katz D., Werthel J.D., Sekaran P., Amaravathi R.S., Valenti P. (2017). The main cause of instability after unconstrained shoulder prosthesis is soft tissue deficiency. J. Shoulder Elb. Surg..

[28] Kany J., Amouyel T., Flamand O., Katz D., Valenti P. (2015). A convertible shoulder system: Is it useful in total shoulder arthroplasty revisions?. Int. Orthop..

[29] Boileau P., Avidor C., Krishnan S.G., Walch G., Kempf J.F., Molé D. (2002). Cemented polyethylene versus uncemented metal-backed glenoid components in total shoulder arthroplasty: A prospective, double-blind, randomized study. J. Shoulder Elb. Surg..

[30] Magosch P., Lichtenberg S., Tauber M., Martetschläger F., Habermeyer P. (2021). Prospective midterm results of a new convertible glenoid component in anatomic shoulder arthroplasty: A cohort study. Arch. Orthop. Trauma Surg..

[31] Magosch P., Habermeyer P., Vetter P. (2021). Radiologic midterm results of cemented and uncemented glenoid components in primary osteoarthritis of the shoulder: A matched pair analysis. Arch. Orthop. Trauma Surg..

[32] Castagna A., Randelli M., Garofalo R., Maradei L., Giardella A., Borroni M. (2010). Mid-term results of a metal-backed glenoid component in total shoulder replacement. J. Bone Jt. Surg..

[33] Keener J.D., Wei A.S., Kim H.M., Steger-May K., Yamaguchi K. (2009). Proximal humeral migration in shoulders with symptomatic and asymptomatic rotator cuff tears. J. Bone Jt. Surg..

[34] Montoya F., Magosch P., Scheiderer B., Lichtenberg S., Melean P., Habermeyer P. (2013). Midterm results of a total shoulder prosthesis fixed with a cementless glenoid component. J. Shoulder Elb. Surg..

[35] Ammarullah M.I., Afif I.Y., Maula M.I., Winarni T.I., Tauviqirrahman M., Jamari J. (2022). Tresca stress evaluation of Metal-on-UHMWPE total hip arthroplasty during peak loading from normal walking activity. Mater. Today Proc..

[36] Jamari J., Ammarullah M.I., Saad A.P.M., Syahrom A., Uddin M., van der Heide E., Basri H. (2021). The Effect of Bottom Profile Dimples on the Femoral Head on Wear in Metal-on-Metal Total Hip Arthroplasty. J. Funct. Biomater..

